# Association between surgical volumes and postoperative outcomes after gastric cancer resection

**DOI:** 10.1186/s13037-026-00505-y

**Published:** 2026-08-04

**Authors:** Marie Klein, Rene Warschkow, Yanic Ammann, Kristijan Ukegjini, Patrick Folie, Bruno Schmied, Ignazio Tarantino

**Affiliations:** Department of General, Visceral, Endocrine and Transplant Surgery, HOCH - Cantonal Hospital of St. Gallen, Rorschacherstrasse 95, St. Gallen, CH-9007 Switzerland

**Keywords:** Hospital volume, Mortality, Complication, Length of hospital stay, Gastric cancer resection, Failure to rescue

## Abstract

**Background:**

The association between hospital volume and outcomes after gastric cancer resection remains controversial. This Swiss national registry study investigated the association between annual hospital-volume for gastric cancer resection and short-term outcomes.

**Methods:**

This retrospective population-based study included adult patients undergoing elective gastric cancer resection in Switzerland between 2013 and 2022. Annual hospital volume was defined as the number of gastric cancer resections performed at the treating hospital in the calendar year of surgery. The primary endpoint was in-hospital mortality. Secondary endpoints were postoperative complications, failure to rescue (FTR), reoperation, and length of hospital stay (LOS). Generalized additive regression models (GAM), standardized mortality ratios (SMR), quantile regression, and trend tests were used to assess the association with hospital volume.

**Results:**

Among 3,861 gastric cancer resections, in-hospital mortality was 3.1%, complications occurred in 48.0%, and mean LOS was 17.1 days. Higher annual hospital volume was associated with lower mortality rates (odds ratio per 10 additional resections (OR): 0.769, 95% confidence interval (CI) 0.617–0.957; *p* = 0.019) and FTR (OR: 0.727, 95% CI: 0.581–0.910; *p* = 0.005), but with higher complication rates (OR: 1.104, CI: 1.027–1.185; *p* = 0.007). The very-high-volume quintile (22–39 resections/year) had the lowest mortality rate (1.4%), despite the highest comorbidity burden and complication rate. Gastric cancer resection volume was associated with SMR (*p* < 0.001). No significant overall association between hospital volume and LOS was observed. During the study period, mortality and FTR decreased, while annual hospital volume increased.

**Conclusion:**

In this Swiss cohort, higher annual gastric cancer resection volume was associated with lower in-hospital mortality and FTR rates. The lower mortality observed in higher-volume hospitals occurred despite a greater comorbidity burden and higher recorded complication rates, suggesting that differences in the response to postoperative complications may contribute to the observed volume–outcome relationship.

**Supplementary Information:**

The online version contains supplementary material available at https://doi.org/10.1186/s13037-026-00505-y.

## Background

Gastric cancer represents the fifth most common malignancy worldwide and the fourth leading cause of cancer-related mortality [1]. In Switzerland, an average of 1057 individuals were diagnosed with gastric cancer annually between 2017 and 2021, accounting for approximately 2% of all newly diagnosed cancers. With a five-year survival rate of 40%, gastric cancer accounts for about 3% of all cancer-related deaths each year in Switzerland [2, 3]. Surgical resection with adequate lymphadenectomy or, in selected early-stage cases, endoscopic resection remains the only curative treatment option. Perioperative chemotherapy and immunotherapy have been shown to reduce recurrence rates and improve overall survival [4]. The management of gastric cancer is complex and requires an experienced multidisciplinary team, surgical expertise, and appropriate infrastructure. Although numerous studies have demonstrated a volume–outcome relationship in complex abdominal oncologic surgery, the evidence specifically addressing gastric cancer resection remains controversial [5–16]. In Switzerland, unlike for other tumor entities such as esophageal, pancreatic, or low rectal cancer, treatment of gastric cancer is not restricted to highly specialized centres meeting defined minimum case volume thresholds and complying with additional criteria regarding infrastructure, availability of specialized clinicians and research, but may be performed in any Swiss hospital. Given the ongoing debate regarding the impact of hospital case volume on outcomes after gastric cancer resection and the absence of centralization policies for gastric cancer surgery in Switzerland, an evaluation of volume–outcome relationships within the Swiss healthcare system may help clarify this association.

This retrospective registry study of the Swiss national data between 2013 and 2022 analyses the association between annual hospital-volume for gastric cancer resections per year and short-term outcomes.

## Methods

This study was approved by the Ethics Committee of Eastern Switzerland (BASEC B2025-00702), registered under ClinicalTrials.gov (NCT07072429), and is compliant with the Strengthening the Reporting of Observational Studies in Epidemiology (STROBE) guidelines [17]. It conforms to the provisions of the Declaration of Helsinki.

### Study design and population

This is a retrospective population-based analysis from the Swiss national inpatient database between 2013 and 2022. The anonymised data were obtained from the Swiss Federal Statistics Office (FSO) via a data contract for one-time delivery and use of individual data (without personal identification) in Switzerland. The dataset contained detailed information from all Swiss hospitals’ inpatient cases between 2013 and 2022 as described in the Supplementary Material. The FSO classifies hospitals into university, tertiary, three levels of basic, and specialised surgical hospitals according to the number of cases per year and a special hospital score assigned by the Swiss Medical Association (FMH). The identification number assigned to each hospital changed annually.

### Case selection

The cases were selected according to their “International Statistical Classification of Diseases and Related Health Problems 10th Revision” (ICD) and “Swiss Classification of Operations” (CHOP) codes [18, 19]. A case was selected for gastric cancer resection when at least one of the ICD codes started with C15.2 (malignant neoplasia of the abdominal oesophagus) or C16 (gastric malignant neoplasia) and at least one of the CHOP codes started with 43.5, 43.6, 43.7, 43.8 or 43.9 (partial or total gastrectomy).

Exclusion criteria included emergency admission, incomplete case documentation, age under 20 years, and hospitalisation in hospitals other than general or surgical hospitals. Incomplete documentation occurs due to the strictly full calendar year (from 1st January to 31st December) based classification of the FSO. Data from patients hospitalised overnight on the 31st of December are therefore incomplete [5]. The inclusion of 18- and 19-year-olds was not possible due to the data structure with five-year age groups.

### Primary and secondary endpoints

The primary endpoint of this study was postoperative mortality, which was defined as in-hospital mortality after the surgical procedure.

Secondary endpoints were intra- and postoperative complications, length of hospital stay (LOS), and failure to rescue (FTR). The analysed intra- and postoperative complications are shown in Table 3.

Hospital volume was defined as the number of gastric cancer resections performed at the treating hospital in the calendar year of each patient’s surgery, resulting in a patient-weighted overall mean hospital volume. This must be distinguished from the annual number of hospitals providing the operation and their caseload. Hospital gastric cancer diagnostic volume was defined as the annual number of patients diagnosed with gastric cancer at each hospital.

Further objectives were to evaluate trends in age, gender, Elixhauser comorbidity index, ICU stay, insurance type, hospital type, and robotic access between 2013 and 2022 [20–22]. 

### Statistical analysis

The R statistical software was used for statistical analysis (www.r-project.org). A two-sided p-value of less than 0.05 was considered statistically significant. Continuous data were expressed as the mean ± standard deviation (SD) and the range. In descriptive analysis, proportions were compared with chi-square statistics, and continuous variables were compared with the t test and the Mann‒Whitney U test, as appropriate. Confidence intervals for proportions were estimated as Agresti-Coull intervals. Adjustment for multiple testing was performed according to Benjamini and Hochberg [23]. 

The associations between the hospital volume and mortality, complications and reoperations were assessed by generalized additive logistic regression models (GAM) adjusting for year of operation and the Elixhauser comorbidity index with random effect B-splines. The confounding variable set included sex (male vs. female), age (continuous), and insurance (general vs. private (semi-private and private)). Results were further elucidated by a marginal effect analysis as described in the Supplementary Material [24, 25]. Hospitals were categorised according to their annual gastric cancer resection volume into equal-sized volume quintiles (very low, low, intermediate, high, and very high) and postoperative outcomes were compared across these volume quintiles using Pearson’s chi-square test [26]. 

Standardized mortality ratios (SMRs) were calculated as the ratio of the observed to expected deaths. The expected mortality was estimated using a GAM logistic regression model based on 64,106 deaths among 6,646,644 elective in-hospital cases among patients aged ≥ 20 years between 2013 and 2022. Expected mortality was predicted based on age (5-year intervals), gender, year of hospitalisation, and the Elixhauser comorbidity score as factorial variables. This model explained 24.5% of the deviance (adjusted R^2^ = 0.05). Differences between observed and expected mortality were assessed using chi-square tests, and associations between annual hospital volume and SMR were evaluated using likelihood-ratio tests in univariable and multivariable poisson regression analyses.

FTR was defined as in-hospital mortality among patients with major complications. Major complications were defined according to the literature and are marked in Table 3 [27, 28]. 

For the LOS, an ordinary least square regression was complemented by an in-depth analysis by quantile regression analysis to assess the associations between the hospital volume and the different lengths of the LOS, not disturbed by outliers or skewed data, as described in the Supplementary Material [29]. 

Time trends were assessed using the non-parametric Mann-Kendall trend test from the R trend library [30]. Its value is between − 1 (perfect descending trend) and + 1 (perfect ascending trend) with 0 representing no trend at all [31]. 

Detailed descriptions of the statistical analyses are provided in the Supplementary Material.

## Results

### Case selection

**Fig. 1:**
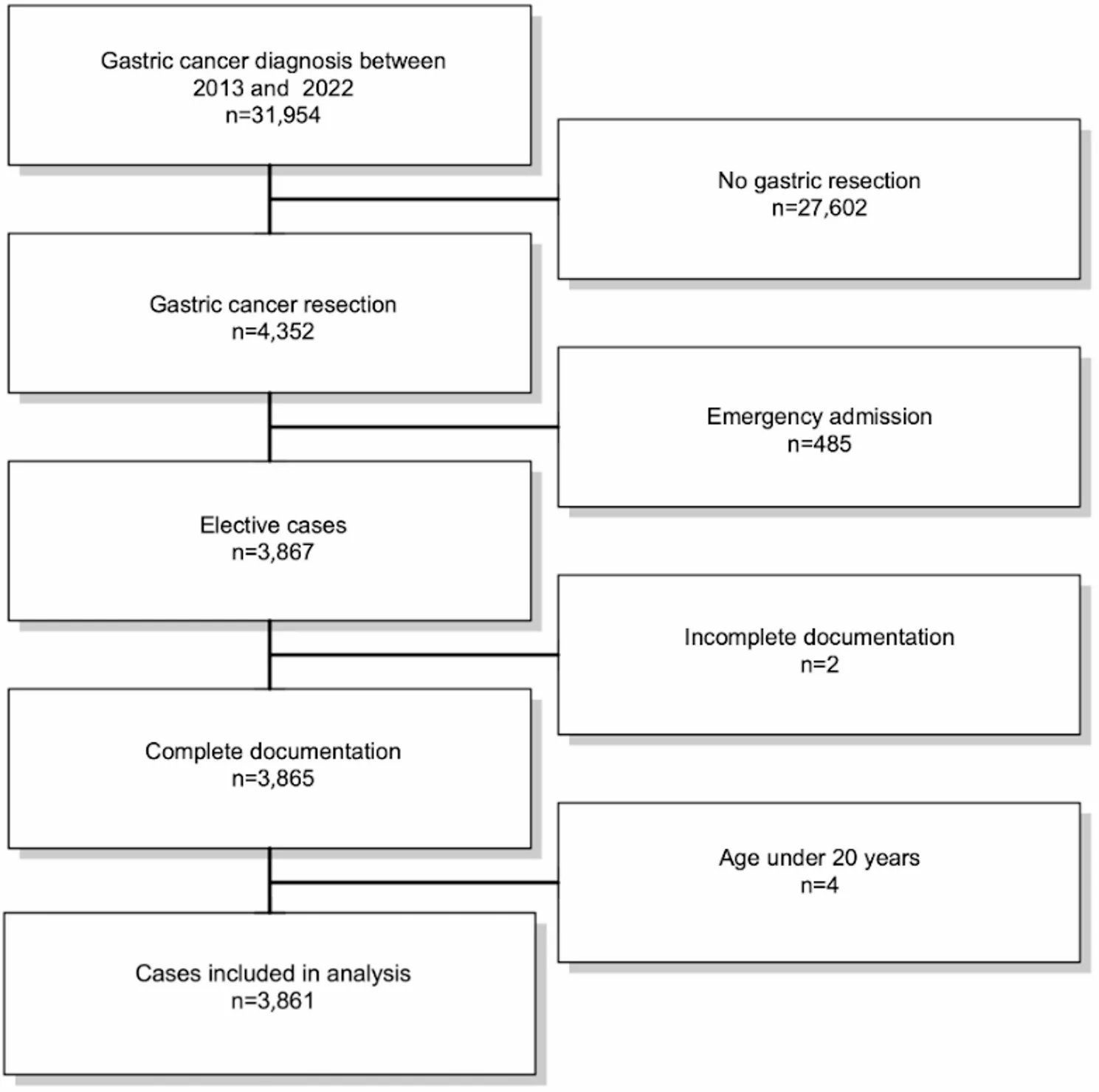
Case selection

The dataset provided by the FSO comprised 14,438,938 cases. Among these, 31’954 cases had a gastric cancer diagnosis, and 4,352 patients underwent gastric cancer resection. A total of 485 cases were excluded due to emergency admission; two cases were excluded due to incomplete case documentation and four patients under the age of 20 years were excluded. Finally, 3,861 cases were included in the analysis (Fig. 1).

### Baseline characteristics and distribution of gastric cancer resection volume

**Fig. 2:**
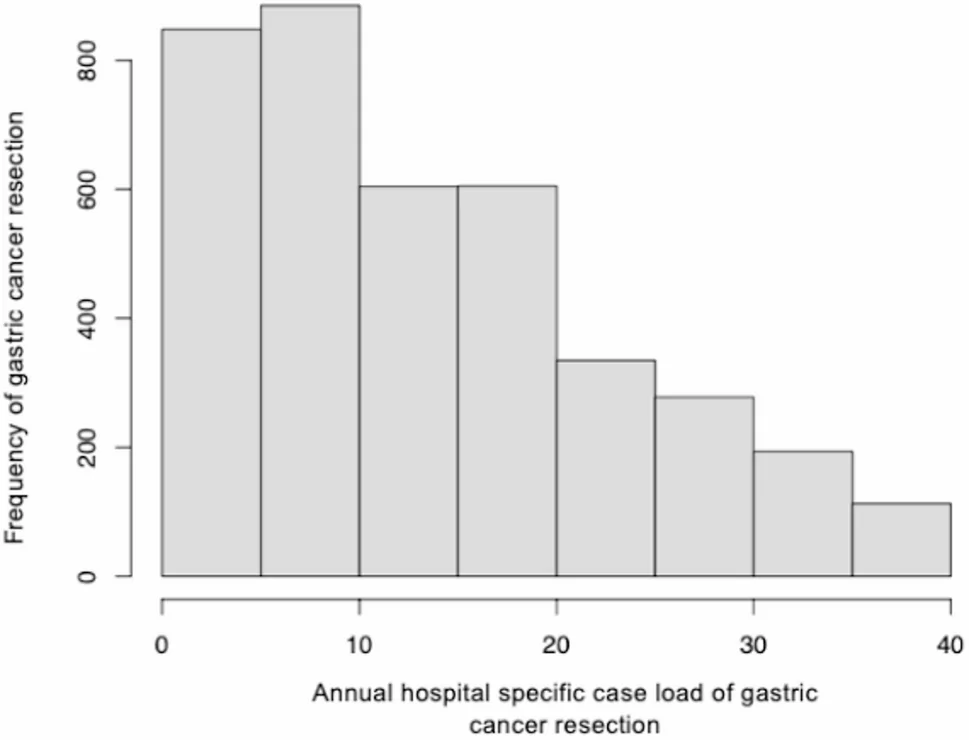
Frequency and distribution of gastric cancer resection according to the hospital volume

Table 1 summarises the baseline characteristics and short-term postoperative outcomes. A total of 3,861 gastric cancer resections were predominantly performed in male patients (64.2%) with a mean age of 66.0 years. Patient’s overall mean Elixhauser comorbidity score was 17.9 ± 9.6 and differed significantly across hospital-volume quintiles, with the highest scores observed in the very-high-volume quintile (19.4 ± 10.2, *p* < 0.001). (Table 2) Each year, 321 to 436 gastric cancer resections were performed in 55 to 67 hospitals, with an average of 6.6 resections per hospital and significant time trends towards more resections in fewer hospitals over time. (Table 4) The mean annual hospital volume was 14.0 ± 9.6, ranging from one to 39, with a median of 12 and an interquartile range from 6 to 19 with a right-skewed distribution (Fig. 2). The annual hospital-volume categories based on quintiles were very low, low, intermediate, high, and very high, corresponding to 1 to 5, 6 to 9, 10 to 15, 16 to 21, and 22 to 39 resections per year. Most gastric cancer resections were performed at tertiary hospitals (2,542, 65.8%), followed by university hospitals (832, 21.5%) and other hospitals (487, 12.6%). Volume categories differed significantly between hospital types (*p* < 0.001), with university hospitals mainly represented in the high- and very-high-volume quintiles (41.0% and 40.3%), tertiary hospitals equally distributed across volume-quintile groups, and hospitals other than university or tertiary hospitals mainly belonging to the very-low-volume quintile group (68.6%). The median volume of 12 resections per year was exceeded in 86.2%, 46.1% and 0% of university, tertiary, and other hospitals, respectively. (Supplementary Material: Table SM1)

Partial gastrectomies were performed more often than total gastrectomies (59.8% versus 40.2%) and equally distributed across volume quintiles. Of the 3,861 gastrectomies, 462 (12.0%) were performed using a minimally invasive approach, with the proportion of both robotic (very-high-volume: 6.5%, *p* = 0.038) and laparoscopic (very-high-volume: 9.2%, *p* < 0.001) procedures increasing across hospital-volume quintiles.

**Table 1:** Case baseline characteristics

Variable	Label	Total *n* = 3861
Age (years)	Mean (SD)	66.0 (12.5)
	Range	20.0–97.0
Sex	Male	2478 (64.2%)
	Female	1383 (35.8%)
Insurance	General	2875 (74.5%)
	Private	986 (25.5%)
Elixhauser comorbidity score	Mean (SD)	17.9 (9.6)
Lymph node metastasis	Yes	1382 (35.8%)
Distant metastasis	Yes	388 (10.0%)
Type of hospital	University	832 (21.5%)
	Tertiary	2542 (65.8%)
	Other	487 (12.6%)
Hospital volume	Mean (SD)	14.0 (9.6)
	Range	1.0–39.0
	≤ 12.0	1972 (51.1%)
	> 12.0 (50th percentile)	1889 (48.9%)
	> 19.0 (75th percentile)	959 (24.8%)
	> 29.0 (90th percentile)	367 (9.5%)
Gastrectomy	Partial	2310 (59.8%)
	Total	1551 (40.2%)
Laparoscopic access	Yes	264 (6.8%)
Robotic access	Yes	198 (5.1%)
ICU stay	Yes	2232 (57.8%)
ICU LOS (hours)	Mean (SD)	81.9 (145.2)
	Range	1.0–1768.0

SD: Standard deviation; ICU: intensive care unit; LOS: Length of stay in the intensive care unit

**Table 2:** Comorbidity and postoperative outcomes stratified by annual gastric cancer resection volume quintiles

	Annual gastric cancer resection volume						
		Very low	Low	Intermediate	High	Very high	
	**Total** (*n* = 3861)	**1 to 5** (*n* = 678)	**6 to 9** (*n* = 706)	**10 to 15** (*n* = 833)	**16 to 21** (*n* = 872)	**22 to 39** (*n* = 772)	**p-value**
Elixhauser comorbidity score, mean (SD)	17.9 (9.6)	16.3 (9.0)	17.9 (9.5)	18.0 (9.6)	17.6 (9.5)	19.4 (10.2)	< 0.001
Mortality	118 (3.1%)	19 (2.8%)	27 (3.8%)	27 (3.2%)	34 (3.9%)	11 (1.4%)	0.030
Overall complications	1855 (48.0%)	294 (43.4%)	316 (44.8%)	398 (47.8%)	432 (49.5%)	415 (53.8%)	< 0.001
Major complications	1595 (41.3%)	240 (35.4%)	268 (38.0%)	351 (42.1%)	372 (42.7%)	364 (47.2%)	< 0.001
Failure to rescue	117 (7.3%)	19 (7.9%)	26 (9.7%)	27 (7.7%)	34 (9.1%)	11 (3.0%)	0.007

Hospitals were stratified into quintiles according to annual gastric cancer resection volume. Elixhauser comorbidity score: mean (SD); all other values: number (%). Failure to rescue was defined as in-hospital mortality among patients with major complications

### Mortality

**Fig. 3:**
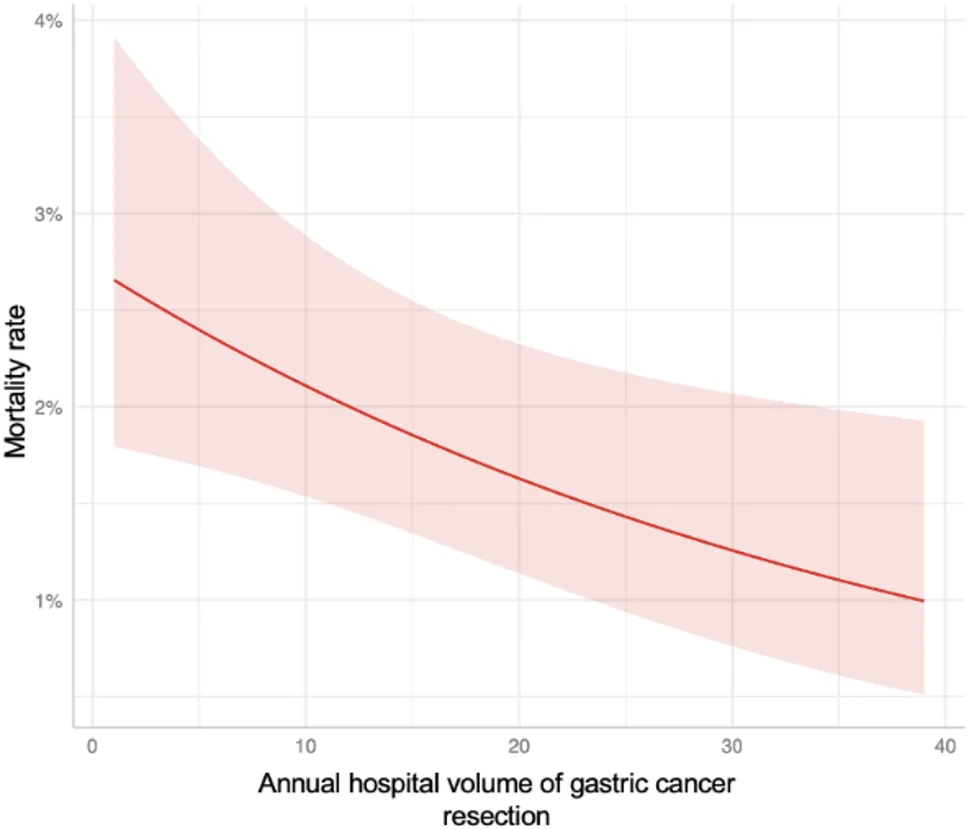
Marginal effect of hospital volume on mortality

Overall mortality after gastric cancer resection was 3.1%. When stratified by hospital-volume quintiles, in-hospital mortality differed significantly across quintiles and was lowest in the very-high-volume quintile (1.4%, *p* = 0.030). (Table 2) In multivariable GAM logistic regression, a significant association between annual hospital volume and mortality was observed (for every ten additional cases: odds ratio (OR) = 0.769, 95% confidence interval (CI): 0.617 to 0.957, *p* = 0.019). (Fig. 3) Higher age (OR = 1.069, CI: 1.047 to 1.091, *p* < 0.001) was independently associated with increased odds of mortality, whereas female sex was associated with lower odds of mortality (OR = 0.586, CI: 0.373 to 0.920, *p* = 0.020). Year of operation (*p* = 0.007) and Elixhauser comorbidity score (*p* < 0.001) were significant random effects.

Overall, 118 deaths were observed versus 164.1 expected deaths, corresponding to an SMR of 0.72 (CI: 0.59 to 0.88, *p* < 0.001), indicating that mortality after elective gastric cancer resection was 28% lower than expected after adjustment for age, sex, year of hospitalisation, and Elixhauser comorbidity scores. Annual gastric cancer resection volume was significantly associated with SMR (*p* < 0.001), primarily reflecting the lower SMR in the very-high-volume quintile compared to other the other volume quintiles (0.35, CI: 0.19–0.63, *p* = 0.008). In multivariable poisson regression, no significant association between SMR and hospital gastric cancer diagnostic volume (*p* = 0.376) or total inpatient volume (*p* = 0.991) was observed. Detailed results of the SMR analysis are provided in the Supplementary Material. (Table SM2)

### Postoperative complications

**Fig. 4:**
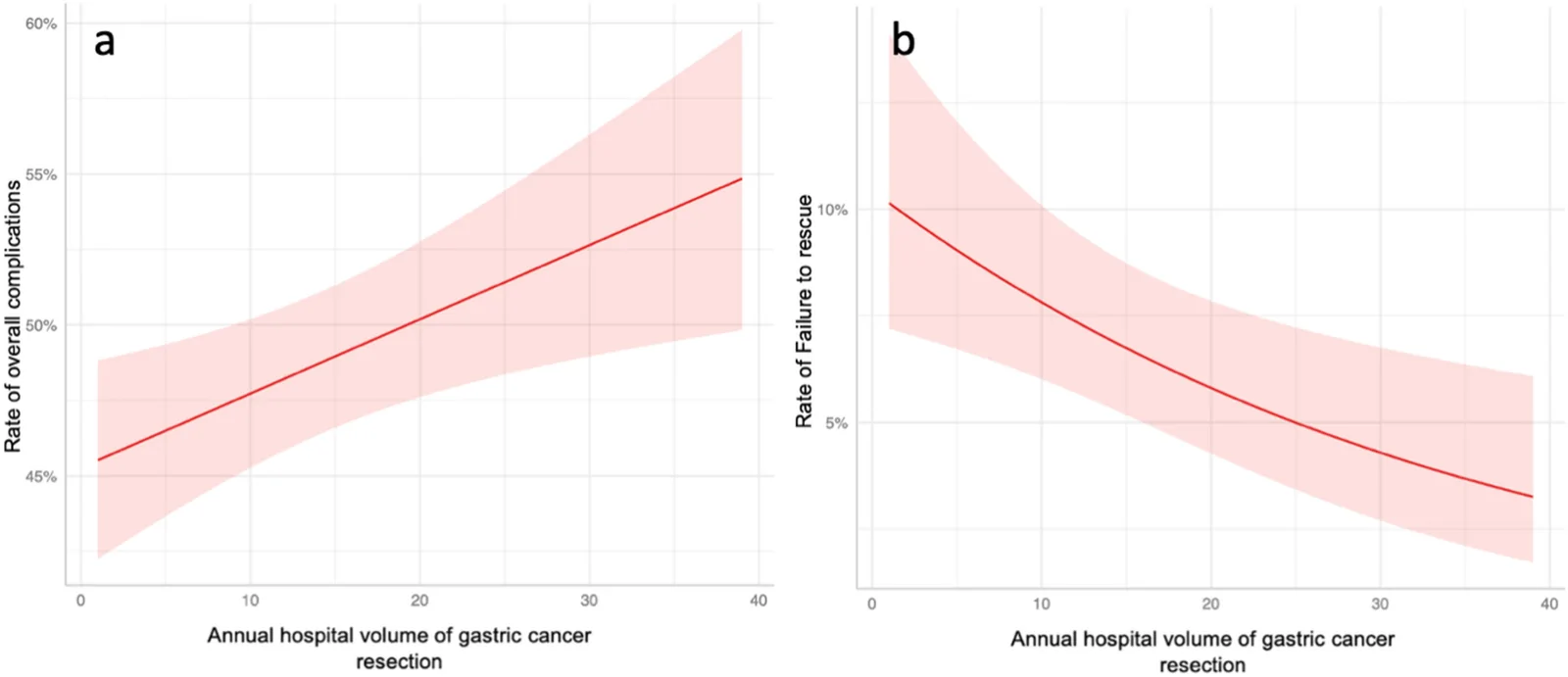
Marginal effect analysis of hospital volume and complications and failure to rescue

The overall complication rate was 48.0% (*n* = 1855), with a major complication rate of 41.3% (*n* = 1595). When stratified by hospital-volume quintiles, overall and major complications rates differed significantly and were highest in the very-high-volume quintile (overall complications: 53.8%, major complications: 47.2%, *p* < 0.001). (Table 2) According to GAM logistic regression, the overall complication rate increased with hospital volume (for every ten additional cases: OR = 1.104, CI: 1.027 to 1.185, *p* = 0.007). (Fig. 4a) Higher age was associated with higher complication rates (OR = 1.019, CI: 1.013 to 1.025, *p* < 0.001). Year of operation (*p* = 0.013) and Elixhauser comorbidity score (*p* < 0.001) were significant random effects. Associations between complications, hospital volume, and mortality according to GAM logistic regression are shown in Table 3.

**Table 3:** Complications

Variable (ICD Code)	Total *n* = 3861	OR hospital volume (95% CI)	*p*-value	OR mortality (95% CI)	*p*-value
Complications overall	1855 (48.0%)	1.104 (1.027–1.125)	**0.007**	-	-
Stroke* (I60, I61, I62, I63, I64)	6 (0.2%)	1.022 (0.440–2.373	0.959	-	-
Coma* (R40)	15 (0.4%)	1.222 (0.740–2.017	0.434	2.481 (0.416–14.786)	0.318
Myocardial infarction* (I21, I22)	21 (0.5%)	0.954 (0.608–1.499)	0.840	8.318 (1.765–39.197)	**0.007**
Cardiac arrest* (I46)	32 (0.8%)	0.776 (0.521–1.157)	0.214	49.608 (14.884–165.34)	**< 0.001**
Arterial fibrillation* (I48)	426 (11.0%)	1.009 (0.883–1.153)	0.893	1.672 (0.877–3.189)	0.119
Pulmonary embolism* (I26.0, I26.9)	101 (2.6%)	0.817 (0.652–1.023	0.078	1.195 (0.38–3.762)	0.760
Deep vein thrombosis (I80.2)	14 (0.4%)	1.079 (0.633–1.839)	0.779	-	-
Pneumonia* (J13-18, J85.1)	301 (7.8%)	0.955 (0.842–1.085)	0.482	2.235 (1.038–4.812)	**0.040**
Pleural effusion* (J90, J91)	489 (12.7%)	1.189 (1.077–1.313)	**< 0.001**	0.683 (0.316–1.477)	0.333
Pleural empyema* (J86.0, J86.9)	72 (1.9%)	1.085 (0.853–1.379)	0.507	4.182 (1.426–12.267)	**0.009**
Aspiration* (J69.0, J69.8, J95.4)	160 (4.1%)	1.083 (0.917–1.279)	0.345	2.142 (0.963–4.766)	0.062
Atelectasis (J98.1)	152 (3.9%)	1.218 (1.036–1.433)	**0.017**	0.095 (0.021–0.421)	**0.002**
Pneumothorax* (J93, J95.80, S27.0)	120 (3.1%)	1.345 (1.124–1.610)	**0.001**	1.603 (0.512–5.019)	0.418
Pulmonary insufficiency, respiratory failure* (J95.1, J95.2, J95.3, J96)	299 (7.7%)	1.353 (1.199–1.528)	**< 0.001**	4.781 (2.342–9.763)	**< 0.001**
ARDS* (J80)	40 (1.0%)	0.846 (0.599–1.195)	0.343	11.286 (3.405–37.407)	**< 0.001**
Acute kidney injury* (N17, N19)	187 (4.8%)	1.152 (0.976–1.358)	0.094	2.258 (1.031–4.943)	**0.042**
Urinary tract infection (N10, N30.0, N30.9, N39.0)	190 (4.9%)	0.801 (0.678–0.947)	**0.009**	1.088 (0.317–3.735)	0.894
Urinary retention (R33)	75 (1.9%)	0.956 (0.749–1.222)	0.722	0.495 (0.084–2.918)	0.438
Anastomotic leakage*^1^ (K91.83)	272 (11.7%)	1.108 (0.976–1.258)	0.114	2.191 (1.003–4.789)	**0.049**
Gastrointestinal ulcer* (K25, K28)	57 (1.5%)	0.927 (0.699–1.229)	0.597	0.698 (0.107–4.548)	0.707
Peritonitis and intraabdominal abscess* (K65)	154 (4.0%)	0.798 (0.665–0.959)	**0.016**	1.364 (0.536–3.475)	0.515
Delayed gastric emptying* (K31.88)	67 (1.7%)	1.096 (0.853–1.407)	0.473	1.842 (0.448–7.567)	0.397
Ileus* (K56, K91.3)	205 (5.3%)	0.880 (0.755–1.027)	0.106	1.307 (0.548–3.114)	0.546
Mesenteric ischemia* (K55)	30 (0.8%)	1.079 (0.746–1.560)	0.686	1.785 (0.318–10.016)	0.51
Bleeding* (T81.0, S36.81)	202 (5.2%)	1.112 (0.961–1.287)	0.153	1.123 (0.427–2.955)	0.814
SIRS* (R65)	164 (4.2%)	0.658 (0.543–0.797)	**< 0.001**	5.277 (2.217–12.564)	**< 0.001**
Shock* (T81.1, R57)	161 (4.2%)	0.936 (0.784–1.116)	0.459	1.044 (0.435–2.504)	0.923
Lymphatic fistula (I89.8, J94.0)	30 (0.8%)	1.416 (1.002–2.002)	**0.048**	-	-
Wound dehiscence* (T81.3)	118 (3.1%)	0.870 (0.710–1.067)	0.180	1.875 (0.719–4.887)	0.199
SSI* (T81.4, T89.02, L08.9)	192 (5.0%)	0.786 (0.666–0.927)	**0.004**	0.843 (0.322–2.206)	0.727
Other vascular complication (T81.7)	5 (0.1%)	0.567 (0.184–1.743)	0.322	-	-
Injury to the lungs or pleura (S27.0, S21.3, S27.6)	52 (1.3%)	1.187 (0.904–1.560)	0.217	1.258 (0.180–8.786)	0.817
Injury to the diaphragm (S27.81)	8 (0.2%)	1.268 (0.648–2.480)	0.488	-	-
Other intraoperative injuries (T81.2)	26 (0.7%)	0.803 (0.518–1.247)	0.329	0.707 (0.047–10.716)	0.803
Intraoperative retained foreign body (T81.5, T81.6)	4 (0.1%)	0.828 (0.280–2.447)	0.733	-	-
Other complications (J95.8, J95.9, K91.88, K91.9, T81.8, T81.9)	314 (8.1%)	1.138 (1.012–1.280)	**0.031**	0.621 (0.212–1.823)	0.386

OR hospital volume (95% CI): Odds ratio for every 10 additional cases according to GAM regression (95% Confidence Interval); OR mortality (95% CI): Odds ratio for mortality according to GAM logistic regression (95% Confidence Interval); DVT: Deep venous thrombosis; ARDS: Acute respiratory distress syndrome; SIRS: Systematic Inflammatory Response Syndrome; SSI: Surgical site infection; *Major complications, ^1^ Data on Anastomotic leakage were only available for the years 2015–2022, the reported percentage is restricted to this period

### Failure to rescue

Of the 118 in-hospital deaths (3.1%), 117 (3.0%) were associated with major complications, the overall FTR rate was 7.3%. When stratified by hospital-volume quintiles, FTR rates differed significantly and were lowest in the very-high-volume quintile (3.0%, *p* = 0.007). (Table 2) According to GAM logistic regression, the FTR rate decreased with hospital volume (for every ten additional cases: OR = 0.727, CI: 0.581 to 0.910, *p* = 0.005). (Fig. 4b) Higher age was associated with higher FTR rates (OR = 1.056, CI: 1.034 to 1.078, *p* < 0.001). Year of operation (*p* = 0.011) and Elixhauser comorbidity score (*p* < 0.001) were significant random effects.

### Length of hospital stay

**Fig. 5:**
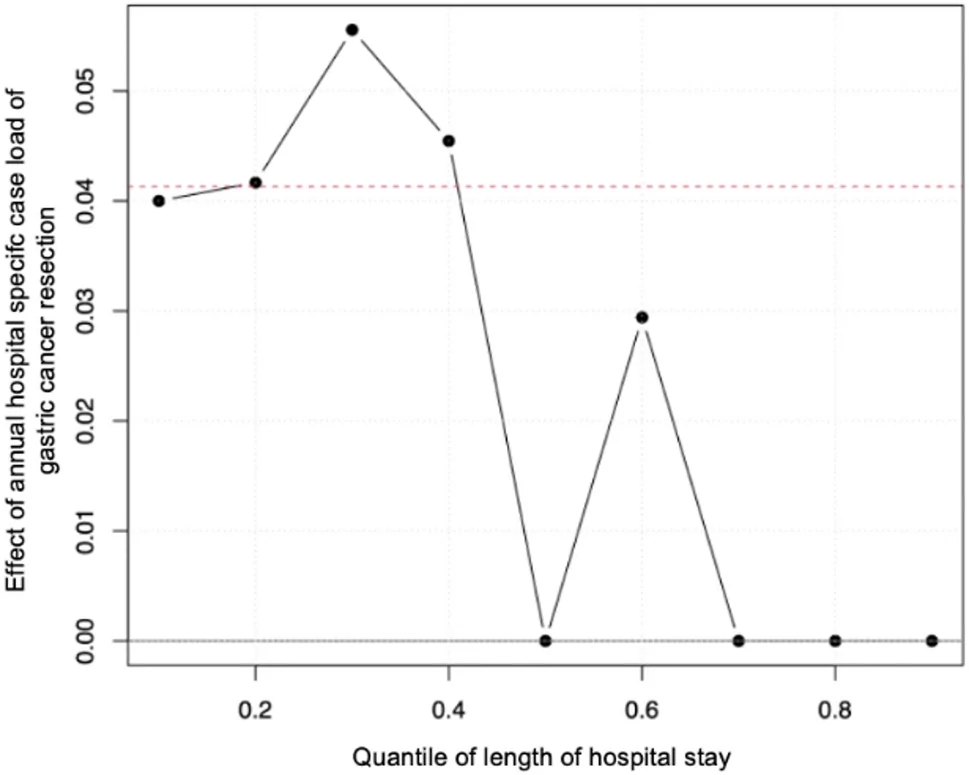
Quantile regression for the effect of the hospital volume on LOS

The mean LOS was 17.1 days. In ordinary regression, no significant association between LOS and hospital volume was observed (β = 0.041, CI -0.001 to 0.084, *p* = 0.058). Quantile regression showed positive point estimates across the lower quantiles (0.1 to 0.4: β = 0.040 to 0.056), reaching statistical significance at the 0.3 quantile (β = 0.056, CI 0.030 to 0.129); coefficients at the 0.5 quantile and above were at or near zero. The joint test of equality of the quantile slopes was significant (*p* < 0.001) showing a heterogeneous effect across the LOS distribution. (Fig. 5)

### Trend analysis between 2013 and 2022

Trend analysis is shown in Table 4. There was a significant reduction in mortality (tau=-0.689, *p* = 0.006) between 2013 and 2022. The total number of operations (tau = + 0.809, *p* < 0.001) and mean hospital volume (tau = + 0.822, *p* < 0.001) increased over time. While there was no significant trend in overall or major complication rates, the reoperation rate increased (tau = + 0.689, *p* = 0.006) and the FTR rate decreased over time (tau =-0.644, *p* = 0.009).

Over the study period, there was a significant increase in the number of cases in the very-high-volume quintile (tau = 0.629, *p* = 0.012), and a significant shift in the distribution of cases towards higher hospital-volume quintiles was observed (Spearman rank correlation rho = 0.236, *p* < 0.001). (Table SM 3)

**Table 4:** Trend analysis

Variable	Sum / average	2013	2022	tau trend	p value*
Number of operations (n)	3861	359	436	0.809	**<0.001**
Number of hospitals (n)	58.8	67	56	-0.552	**0.030**
Mean hospital volume^1^ (n)	14.0	10.3	17.2	0.822	**<0.001**
Mean annual gastric cancer resections per hospital (n)	6.6	5.4	7.8	0.822	**<0.001**
Very-high-volume quintile (%)	19.99	6.41	34.63	0.629	**0.012**
Mean age (years)	66.01	66.99	65.66	-0.111	0.655
Mean Elixhauser comorbidity index (score)	17.85	16.13	18.40	0.778	**0.002**
Women (%)	35.82	34.26	35.32	-0.289	0.245
ICU stay (%)	57.81	67.13	48.39	-0.689	**0.006**
Private insurance (%)	25.54	24.51	21.56	-0.289	0.245
Robotic access (%)	5.13	1.11	12.16	0.778	**0.002**
Mortality (%)	3.06	4.46	2.98	-0.689	**0.006**
Overall complications (%)	48.04	48.47	47.94	0.200	0.929
Major complications (%)	41.31	39.00	42.89	+0.333	0.180
Failure to rescue (%)	7.34	10.71	6.95	-0.674	**0.007**
Reoperation (%)	7.82	3.62	8.26	0.689	**0.006**
Mean LOS (days)	17.1	20.4	14.6	-0.778	**0.002**

*Significant values are bold

^1^ Hospital volume was defined as the number of gastric cancer resections performed at the treating hospital in the calendar year of each patient’s surgery, resulting in a patient-weighted overall mean hospital volume.

ICU: Intensive care unit; LOS: Length of hospital stay

### Sensitivity analysis

A sensitivity analysis was performed including only patients undergoing total gastrectomy and excluding partial gastrectomies: A total of 1,551 total gastrectomies were performed, and the mean hospital volume for total gastrectomies was 8.2 per year. According to logistic GAM regression, higher hospital volume was associated with lower point estimates for mortality (OR = 0.809, CI: 0.415 to 1.576) and FTR (OR = 0.726, CI: 0.362 to 1.456), but nether association reached statistical significance; the point estimates were directionally consistent but had wide confidence intervals.

## Discussion

The present retrospective Swiss national registry study investigated the association between hospital volume and short-term postoperative outcomes after gastric cancer resections in Switzerland. The majority of gastric cancer resections were performed in tertiary hospitals, the mean annual hospital volume was 14 cases, ranging from 1 to 39 resections per year. The postoperative mortality rate was 3.1%, and the overall complication rate was 48.0%, with a FTR rate of 7.3%.

Mortality and FTR decreased significantly with increasing hospital volume even though comorbidity burden and complication rates increased with hospital volume, especially in the very-high-volume quintiles. These findings indicate that the association between hospital volume and mortality depends more on the ability to recognize and manage postoperative complications than on the occurrence of postoperative complications alone. However, differences in postoperative surveillance, documentation, and coding practices may also have contributed to the higher recorded complication rates observed in higher-volume centers. The overall complication rate in our cohort was higher than rates reported in previous studies [32–34]. As coding of complications is relevant for reimbursement in Switzerland, more complete documentation of postoperative complications may have contributed to this difference. Despite these differences in complication rates, our results are consistent with two German studies showing lower mortality and FTR rates at higher-volume hospitals alongside similar complication rates, and with the recent study by Gerber et al. reporting lower FTR rates despite higher major complication rates at higher-volume hospitals. In the SMR analysis, annual gastric cancer resection volume, but not hospital gastric-cancer diagnostic volume or total inpatient volume, was associated with standardized mortality. This may suggest that procedure-specific experience may be particularly relevant for short-term outcomes after gastrectomy in our study cohort.

The appropriate minimum annual hospital volume for gastric cancer resections is still a matter of debate. In the present study, the lowest mortality and FTR rates were observed in the very-high-volume quintile (22 to 39 gastric cancer resections per year), while the high-volume quintile (16 to 21 gastric cancer resections per year) did not show similarly favourable outcomes. This is consistent with the findings of a German study comparing hospitals with higher caseloads (approximately 29 gastric cancer resections per year) with hospitals with a very low caseload (< 2 gastric cancer resections per year), reporting a 50% reduction in in-hospital mortality in higher-volume centres; similarly, a Dutch study found improved outcomes in hospitals performing at least 31 gastric cancer resections annually compared with fewer than ten [12, 13]. With regard to lower case volumes, a previous Swiss study comparing hospitals performing more than ten versus fewer than ten gastric cancer resections annually found no significant difference in mortality (> 10 gastric cancer resections: 1%, < 10 gastric cancer resections: 2%, *p* = 0.581) [14]. 

A recent study by Gerber et al. about the effect of hospital volume on gastric cancer resection outcomes in Switzerland reported a similar volume-mortality relationship: in-hospital mortality ranged from 4.6% in the lowest-volume group to 2.2% in centres performing more than 20 annual gastric cancer resections, which is in line with our findings. In contrast to our results, Gerber et al. found that FTR was associated with inpatient volume and hospital type, but not with gastric cancer resection volume. These differences may be explained by methodological and case-selection variation: Gerber et al. analysed data from 1998 to 2021 and included surgical, local, and endoscopic resections; our analysis was limited to elective gastrectomies performed from 2013 to 2022. Gerber et al. used volume categories based on the distribution in the final five study years and mixed-effects spline models with hospital-level random intercepts. We used patient-level annual hospital volume, categorized hospitals into quintiles, and applied GAM models adjusted for age, sex, Elixhauser comorbidity scores, insurance status, and year of surgery. Differences in the definitions of severe complications and failure to rescue may also have contributed [16]. 

In the present study, the Elixhauser comorbidity scores increased significantly between 2013 and 2022, despite no change in mean age, indicating a greater coded comorbidity burden among patients undergoing gastric cancer resections over time, which is consistent with the results of a national cohort study on patients undergoing total gastrectomy [35]. However, mortality and FTR rates decreased significantly during the study period, while overall and major complication rates were stable and reoperation rates increased. These findings indicate that patients who developed postoperative complications were less likely to die during the study period, which may be explained by earlier recognition and more aggressive management of postoperative complications, including a lower threshold for reoperation. Similar observations were reported in Dutch and German studies, where mortality and FTR after gastric cancer resections decreased over time [36, 37]. 

Minimally invasive gastric cancer resections were more frequently performed in higher hospital-volume quintiles in the present cohort, and robotic approach increased over time. This is consistent with a study using data from US National Cancer Database showing that minimally invasive gastrectomy has increased over time but is less frequently used in lower-volume hospitals [38]. This pattern may be explained by the procedure-specific expertise required for minimally invasive gastrectomies, which could contribute to their more frequent use in higher-volume centres [39]. 

Hospital volume was associated with a tendency towards longer LOS in the lower half of the LOS distribution. This pattern may reflect the tendency of high-volume hospitals to manage more complex cases, as shown by the significant increase in Elixhauser comorbidity scores with increasing annual gastric cancer resection volume, thereby reducing the likelihood of very short inpatient stays. The association between hospital volume and LOS was no longer observed in the upper half of the LOS distribution. Higher LOS quantiles are more likely to include patients with postoperative complications; as hospitals with higher caseloads gain experience in managing complications, improved treatment of complications may offset the inherently more complex cases in terms of LOS for higher quantiles. Trend analysis of the Swiss national data showed that LOS was reduced over time and that patients were less likely to stay in the ICU. This may be explained by the widespread implementation of the Enhanced Recovery After Surgery (ERAS) after gastrectomy within the last few years, which has been shown to significantly reduce LOS, as well as by the increase in robotic gastric cancer resections, which have been associated with shorter LOS than open gastrectomy [40–42]. 

Our results demonstrate a decreasing number of hospitals performing gastric cancer resections in Switzerland and a shift in the distribution of cases towards higher-volume hospitals over recent years, suggesting an ongoing centralization of gastric cancer resections in Switzerland. A recent meta-analysis examining the relationship between hospital surgical case volume and postoperative mortality after gastrectomy for gastric cancer showed a steep decline from 17.7% to 0.3% until the hospital volume reached a plateau of 100 gastric cancer resections per year, suggesting this threshold as the definition of a high-volume center [15]. Given the overall small case number in Switzerland, no hospital in our cohort reached such high annual volumes. Applying a threshold of 100 annual gastric cancer resections per year would restrict gastric cancer surgery to only three hospitals nationwide. To date, no international consensus exists regarding a minimum case volume required to define a high-volume gastric cancer resection center, and most thresholds have been arbitrarily selected [43]. When advocating for further centralization of gastric cancer resections in Switzerland, the relatively small national caseload and geographical considerations must be acknowledged. Establishing higher-volume centers would substantially reduce the number of hospitals performing gastric cancer resections, potentially increasing patient travel distances, limiting proximity to family support, and complicating postoperative continuity of care and long-term follow-up. A US study demonstrated that most patients undergoing gastric cancer resection selected the nearest hospital, and hospital choice was not primarily driven by outcome data [44]. Alongside further centralization, collaborative networks between lower- and higher-volume hospitals may represent a pragmatic alternative. Such networks could facilitate multidisciplinary case review, shared expertise, and may be particularly appropriate for referral of patients with increased operative risk due to substantial comorbidities or complex disease to high-volume centers. This approach could enable vulnerable patients to benefit from the experience and resources of high-volume hospitals while preserving local access to preoperative assessment, postoperative care, and long-term follow up.

Several limitations of the present study warrant consideration: As an observational registry-based study, causal inferences cannot be drawn. Residual confounding due to unmeasured variables is likely, particularly since FSO data were not originally collected for research purposes and lack detailed clinical information such as ASA classification, tumour histology, lymph node yield as an established quality indicator for oncologic gastric cancer resections, Clavien–Dindo grading of complications, and individual surgeon volume. The conversion of clinical information into administrative codes introduces the risk of miscoding, underreporting, and incomplete documentation and over a nearly ten-year study period, changes in coding and documentation cannot be excluded as a possible confounding factor [45]. The SMR analysis was standardized for age, sex, year of hospitalisation, and Elixhauser comorbidity scores but other confounders like tumour stage, or surgical complexity were not accounted for. In addition, mortality was limited to deaths occurring in-hospital, this may underestimate postoperative morbidity and mortality, as a relevant proportion of surgery-related deaths occur after hospital discharge [46]. Finally, given the overall small number of gastric cancer resections in Switzerland, particularly at the individual hospital level, this study cannot be generalised to countries with substantially higher caseloads.

## Conclusion

In this nationwide Swiss registry-based study conducted between 2013 and 2022, higher annual gastric cancer resection volume was associated with lower in-hospital mortality and FTR rates. These associations were observed despite a higher comorbidity burden and more recorded postoperative complications in the very-high-volume hospitals. The association between gastric cancer resection volume, but not hospital gastric cancer diagnostic volume or overall inpatient volume, and standardized mortality may point to the relevance of procedure-specific experience for perioperative patient safety. Gastric cancer resections were increasingly performed in higher-volume hospitals during the study period. However, given the relatively low national caseload and Switzerland’s geographical structure, the optimal organisation of gastric cancer resections and appropriate volume threshold in Switzerland require further evaluation.

## Supplementary Information

Below is the link to the electronic supplementary material.

Supplementary Material 1

## Data Availability

The anonymised case data supporting this study’s findings can be requested from the Swiss Federal Statistics Office (FSO).
