# Supplementary material for: Association between surgical volumes and postoperative outcomes after gastric cancer resection
Source: Patient Saf Surg. 2026 Aug 4;20:40. doi: 10.1186/s13037-026-00505-y (PMC13732997; doi:10.1186/s13037-026-00505-y)
Supplement: Supplementary file 1 [file 13037_2026_505_MOESM1_ESM.docx]

**Supplementary material**

**Study design and population**

The data included the anonymized identification number of the hospital, hospital type, primary and secondary diagnoses based on the International Statistical Classification of Diseases and Related Health Problems 10^th^ Revision (ICD), operations according the Swiss Classification of Surgical Interventions (CHOP), sex, age, insurance type, type of admission, nine equivalents of nursing manpower use score (NEMS), Simplified Acute Physiology Severity Score (SAPS), number of hours on the intensive care unit (ICU), reoperation, length of hospital stay, and hospital discharge information including in-hospital mortality. [1–4] To ensure data protection, age was categorised into five-year age groups. The Swiss health insurance system divides inpatient hospital stays into three insurance classes: general, semi-private, and private. Semi-private and private represent supplementary insurances with influence on room type (two-bedroom for semi-private, single room for private), comfort, doctor choice, and hospital choice. The medical care and service are independent of the insurance class. [5]

**Primary and secondary endpoints**

The 30 Elixhauser comorbidities were identified through Quan et al.’s ICD codes and determined via the comorbidity package in R. [6, 7] This transforms the ICD codes into binary data of the relevant comorbidities, their (unweighted) sum scores, and their Elixhauser weighted scores. [8, 9] ICU stay was assumed if the corresponding CHOP code was present (89.13.* in 2013, 99.B7.* afterwards), the number of hours on ICU was>0, NEMS was >0, and SAPS was >0. If the number of hours in the ICU was not stated, it was imputed by linear regression as the sum of the NEMS and the SAPS. If only the NEMS Score was given, the mean of the SAPS was used. If no NEMS was given, the mean of the range according to the CHOP codes for ICU treatment was used.

**Statistical analysis**

*Mortality, complications, and reoperation*

GAM can handle large datasets, accommodate many predictors, including random effects, and is nonparametric. [10] The inference of the effect of the hospital volume was based on the assumption of the hospital volume as a linear predictor and complemented by an alternative model with splines based on univariate adaptive smooths. [11] The parametric fixed and random effect splines were penalised by ridge penalty using the identity matrix based on assumption that the coefficients are independent and identically distributed normal random effects. [12]

*Length of hospital stay*

The quantile regression was performed using the quantreg R library using the algorithm described by Barrodale and Roberts for L1-Regressions. [13] The resulting fitted coefficient Beta for the hospital volume on LOS (β) in the linear regression describes an increase (positive) or decrease (negative) in LOS per additional operation in the hospital. A β of +0.1 means an increase in LOS of 0.1 days for each unit increase in hospital volume. The Khmaladze-Test was used to test the hypothesis that a linear model specification is of the location shift or location-scale shift form. [14] The test is based on the Doob-Meyer Martingale transformation approach proposed by Khmaladze in 1981 for general goodness of fit problems and was adapted to quantile regression by Koenker and Xiao in 2002. [15] The joint test of equality of the quantile slopes (joint p) tests if there is a difference between the quantiles.

**Table SM1** Distribution of gastric cancer resections across annual hospital-volume quintiles by hospital type

|  | **Annual gastric cancer resection volume** | | | | | |
| --- | --- | --- | --- | --- | --- | --- |
|  | **Very low** | **Low** | **Intermediate** | **High** | **Very high** |  |
| **Hospital Type** | 1 to 5  (n = 678) | 6 to 9  (n=706) | 10 to 15  (n=833) | 16 to 21  (n=872) | 22 to 39  (n=772) |  |
| **University** | 15 (1.8) | 37 (4.4) | 104 (12.5) | 341 (41.0) | 335 (40.3) |  |
| **Tertiary** | 329 (12.9) | 590 (23.2) | 655 (25.8) | 531 (20.9) | 437 (17.2) |  |
| **Other** | 334 (68.6) | 79 (16.2) | 74 (15.2) | 0 (0.0) | 0 (0.0) |  |

According to Spearman rank correlation, there was a significant correlation between hospital types and annual gastric cancer resection volume (rho=-0.533, p<0.001)

**Table SM2** Standardized Mortality Rates

|  |  | **n** | **Observed deaths** | **Expected deaths** | **SMR** | **95% CI** | **p-value 0** | **p-value uni** | **p-value multi** |
| --- | --- | --- | --- | --- | --- | --- | --- | --- | --- |
| **Overall** |  | 3861 | 118 | 164.1 | 0.72 | 0.59-0.88 | **<0.001** | - | - |
| **Hospital volume quintiles** | very low | 678 | 19 | 27.9 | 0.68 | 0.43-1.07 | 0.715 | **<0.001** | **<0.001** |
|  | low | 706 | 27 | 31.7 | 0.85 | 0.58-1.24 | 0.329 |  |  |
|  | intermediate | 833 | 27 | 38.3 | 0.70 | 0.48-1.03 | 0.715 |  |  |
|  | high | 872 | 34 | 34.6 | 0.98 | 0.70-1.38 | 0.270 |  |  |
|  | very high | 772 | 11 | 31.6 | 0.35 | 0.19-0.63 | **0.008** |  |  |
| **Hospital diagnostic volume quintiles** | very low | 757 | 25 | 32.6 | 0.77 | 0.52-1.13 | 0.784 | **0.003** | 0.376 |
|  | low | 768 | 30 | 32.5 | 0.92 | 0.65-1.32 | 0.337 |  |  |
|  | intermediate | 782 | 25 | 33.6 | 0.74 | 0.50-1.10 | 0.784 |  |  |
|  | high | 764 | 17 | 31.2 | 0.54 | 0.34-0.88 | 0.337 |  |  |
|  | very high | 790 | 21 | 34.0 | 0.62 | 0.40-0.95 | 0.784 |  |  |
| **Total inpatient volume** | very low | 771 | 25 | 32.7 | 0.76 | 0.52-1.13 | 0.925 | **0.009** | 0.991 |
|  | low | 746 | 25 | 30.3 | 0.83 | 0.56-1.22 | 0.925 |  |  |
|  | intermediate | 792 | 23 | 33.3 | 0.69 | 0.46-1.04 | 0.925 |  |  |
|  | high | 777 | 21 | 33.3 | 0.63 | 0.41-0.97 | 0.925 |  |  |
|  | very high | 775 | 24 | 34.4 | 0.70 | 0.47-1.04 | 0.925 |  |  |

SMR: Standardized Mortality Ratio, predicted on age (5-year intervals), gender, year of hospitalisation and Elixhauser comorbidity scores as factorial variables; 95% CI: 95% Confidence interval

Hospital volume: number of gastric cancer resections performed at the treating hospital in the calendar year of each patient’s surgery (patient-weighted)

Hospital gastric cancer diagnostic volume: annual number of patients diagnosed with gastric cancer at each hospital.

p-value 0, overall SRM: p-value for the hypothesis that the observed number deaths is not different from the expected number of deaths; p-value 0, stratified for volume: p-value for the hypothesis that a certain volume quintile is not different from the other quintiles

p-value uni: estimated by likelihood ratio tests, p-value for the hypothesis that the grouping variable is not associated with the SMR in the univariable setting

p-value multi: estimated by likelihood ratio tests, p-value for the hypothesis that the grouping variable is not associated with the SMR in the univariable setting

very low: 1 to 5 annual gastric cancer resections (n=678), low: 6 to 9 annual gastric cancer resections (n=706), intermediate: 10 to 15 annual gastric cancer resections (n=833), high: 16 to 21 annual gastric cancer resections (n=872), very high: 22 to 39 annual gastric cancer resections (n=72)

**Table SM3** Trend-analysis of annual gastric cancer resection volume

|  | **Annual gastric cancer resection volume** | | | | | |
| --- | --- | --- | --- | --- | --- | --- |
|  | **Very low** | **Low** | **Intermediate** | **High** | **Very high** |  |
| **Year** | 1 to 5  (n = 678) | 6 to 9  (n=706) | 10 to 15  (n=833) | 16 to 21  (n=872) | 22 to 39  (n=772) |  |
| **2013** | 78 (21.7) | 89 (24.8) | 111 (30.9) | 58 (16.2) | 23 (6.4) |  |
| **2014** | 83 (25.9) | 94 (29.3) | 64 (19.9) | 53 (16.5) | 27 (8.4) |  |
| **2015** | 70 (21.0) | 46 (13.8) | 107 (32.1) | 110 (33.0) | 0 (0.0) |  |
| **2016** | 73 (20.6) | 65 (18.4) | 86 (24.3) | 130 (36.7) | 0 (0.0) |  |
| **2017** | 71 (18.7) | 56 (14.7) | 134 (35.3) | 73 (19.2) | 46 (12.1) |  |
| **2018** | 65 (16.0) | 77 (19.0) | 48 (11.9) | 117 (28.9) | 98 (24.2) |  |
| **2019** | 73 (17.3) | 37 (8.7) | 54 (12.8) | 67 (15.8) | 192 (45.4) |  |
| **2020** | 73 (17.6) | 62 (15.0) | 75 (18.1) | 86 (20.8) | 118 (28.5) |  |
| **2021** | 43 (9.9) | 93 (21.3) | 90 (20.6) | 93 (21.3) | 117 (26.8) |  |
| **2022** | 49 (11.2) | 87 (20.0) | 64 (14.7) | 85 (19.5) | 151 (34.6) |  |

According to Spearman rank correlation, there was a significant correlation between annual gastric cancer resection volume-quintiles and years (rho=0.236, p<0.001)

**References**

1.	ICD-10-GM Version 2021, Systematisches Verzeichnis, Internationale statistische Klassifikation der Krankheiten und verwandter Gesundheitsprobleme, 10. Revision, Stand: 18. September 2020: Bundesinstitut für Arzneimittel und Medizinprodukte (BfArM) im Auftrag des Bundesministeriums für Gesundheit (BMG) unter Beteiligung der Arbeitsgruppe ICD des Kuratoriums für Fragen der Klassifikation im Gesundheitswesen (KKG); 2020 [Available from: www.bfarm.de – Kodiersysteme – Services - Downloads – ICD-10-GM – Version 2021 accessed 19.04.2025.

2.	Schweizerische Operationsklassifikation (CHOP), Systematisches Verzeichnis - Version 2025: Bundesamt für Statistik; 2024 [Available from: https://www.bfs.admin.ch/bfs/de/home/statistiken/kataloge-datenbanken.assetdetail.32128591.html accessed 19.04.2025.

3.	Miranda DR, Moreno R, Iapichino G (1997) Nine equivalents of nursing manpower use score (NEMS). Intensive Care Medicine 23:760–765. https://doi.org/10.1007/s001340050406

4.	Le Gall J-R (1993) A New Simplified Acute Physiology Score (SAPS II) Based on a European/North American Multicenter Study. JAMA 270:2957. https://doi.org/10.1001/jama.1993.03510240069035

5.	Bundesgesetz über die Krankenversicherung (KVG): Die Bundesversammlung der Schweizerischen Eidgenossenschaft; 1994 [Available from: https://www.fedlex.admin.ch/eli/cc/1995/1328_1328_1328/de accessed 01.01.2025.

6.	Quan H, Sundararajan V, Halfon P, et al (2005) Coding Algorithms for Defining Comorbidities in ICD-9-CM and ICD-10 Administrative Data: Medical Care 43:1130–1139. https://doi.org/10.1097/01.mlr.0000182534.19832.83

7.	Gasparini A (2018) comorbidity: An R package for computing comorbidity scores. JOSS 3:648. https://doi.org/10.21105/joss.00648

8.	Sharma N, Schwendimann R, Endrich O, et al (2021) Comparing Charlson and Elixhauser comorbidity indices with different weightings to predict in-hospital mortality: an analysis of national inpatient data. BMC Health Serv Res 21:13. https://doi.org/10.1186/s12913-020-05999-5

9.	Van Walraven C, Austin PC, Jennings A, et al (2009) A Modification of the Elixhauser Comorbidity Measures Into a Point System for Hospital Death Using Administrative Data. Medical Care 47:626–633. https://doi.org/10.1097/MLR.0b013e31819432e5

10.	Li Z, Wood SN (2020) Faster model matrix crossproducts for large generalized linear models with discretized covariates. Stat Comput 30:19–25. https://doi.org/10.1007/s11222-019-09864-2

11.	Wood SN (2013) On p-values for smooth components of an extended generalized additive model. Biometrika 100:221–228. https://doi.org/10.1093/biomet/ass048

12.	Wilson M, Welch J, Schuur J, et al (2014) Hospital and Emergency Department Factors Associated With Variations in Missed Diagnosis and Costs for Patients Age 65 Years and Older With Acute Myocardial Infarction Who Present to Emergency Departments. Academic Emergency Medicine 21:1101–1108. https://doi.org/10.1111/acem.12486

13.	Koenker RW, D’Orey V (1987) Algorithm AS 229: Computing Regression Quantiles. Applied Statistics 36:383. https://doi.org/10.2307/2347802

14.	Khmaladze EV (1982) Martingale Approach in the Theory of Goodness-of-Fit Tests. Theory Probab Appl 26:240–257. https://doi.org/10.1137/1126027

15.	Koenker R, Xiao Z (2002) Inference on the Quantile Regression Process. Econometrica 70:1583–1612. https://doi.org/10.1111/1468-0262.00342
